# Risk factors for seizure reoccurrence after withdrawal from antiepileptic drugs in individuals who have been seizure-free for over 2 years

**DOI:** 10.1371/journal.pone.0181710

**Published:** 2017-08-01

**Authors:** XingHua Tang, Peimin Yu, Ding Ding, Yan Ge, Yunbo Shi, Ping Wang, Guoxing Zhu, Zhen Hong

**Affiliations:** Institute of Neurology, Huashan Hospital, Fudan University, Shanghai, China; University of Rome Tor Vergata, ITALY

## Abstract

**Purpose:**

To observe risk factors for recurrence after withdrawal from antiepileptic drugs.

**Methods:**

We assessed 1282 patients with a definite diagnosis of epilepsy.

**Results:**

In total, 292 patients between 14 and 80 years of age were grouped according to risk factors for recurrence. Of these individuals, 119 discontinued AED(s) and relapsed. The relapse rate was 34.4 per 100 person-years. We used a Cox regression for multivariate analysis to investigate the influence of the following on seizure recurrence: receiving more than one type of AED (HR = 2.53, 95% CI 1.24–5.16) and more than 6 months prior to initiation of AED treatment (HR 1.47, 95% CI = 1.004–2.15).

**Conclusions:**

Although the decision to discontinue AED treatment necessitates an individual evaluation of each patient, our study suggests that there may be a high risk of recurrence in individuals who: were receiving more than one AEDs and had initiated their AED treatment more than 6 months after the initial appearance of epilepsy symptoms.

## 1. Introduction

Epilepsy is a chronic disease often requiring lifelong treatment. Although 60–70% [1,2,3,4] of newly diagnosed patients remain seizure-free after initiating antiepileptic drug (AED) treatment, approximately 50% of patients have long-term adverse reactions to their medications. Adverse behavioral and cognitive effects have been well described in patients receiving AEDs, and these symptoms have been shown to improve after drug withdrawal[5,6]. People with epilepsy may hope to terminate their daily AED treatment because of 1) concerns about the side effects of the medications, 2) the desire to feel ‘‘cured,” and 3) the inconvenience associated with daily medication[7]. Many epilepsy medications have extended treatment periods, leading to reduced compliance in patients who are receiving long-term treatment, as well as declining drug retention rates, even in seizure-free patients. Considering the adverse long-term effects of AEDs, together with the psychosocial and economic burden of epilepsy, AED withdrawal may be considered after patients are seizure-free for an extended period of time.

However, patients have a high risk of seizure recurrence after drug withdrawal. In a small prospective study of adult patients, relapse occurred in 57% of the group (42.8% did not relapse) during a 4-year discontinuation program[8]. Several factors are thought to influence seizure recurrence after drug withdrawal: age at seizure onset, duration of active disease, number of years of seizure remission[8], a history of treatment with multiple AEDs[9], hippocampal atrophy[10], partial onset seizures, the presence of multiple types of seizures, focal epileptiform abnormalities on (electroencephalography) EEG readings, and increased EEG abnormalities during or after AED discontinuation[11]. However, there are few data regarding patients who have withdrawn from AEDs in China, especially among adults and young people.

We used a retrospective study design to identify factors affecting reoccurrence of seizures following drug withdrawal in Chinese epilepsy patients aged 14 years and older.

## 2. Patients and methods

**Fig 1:**
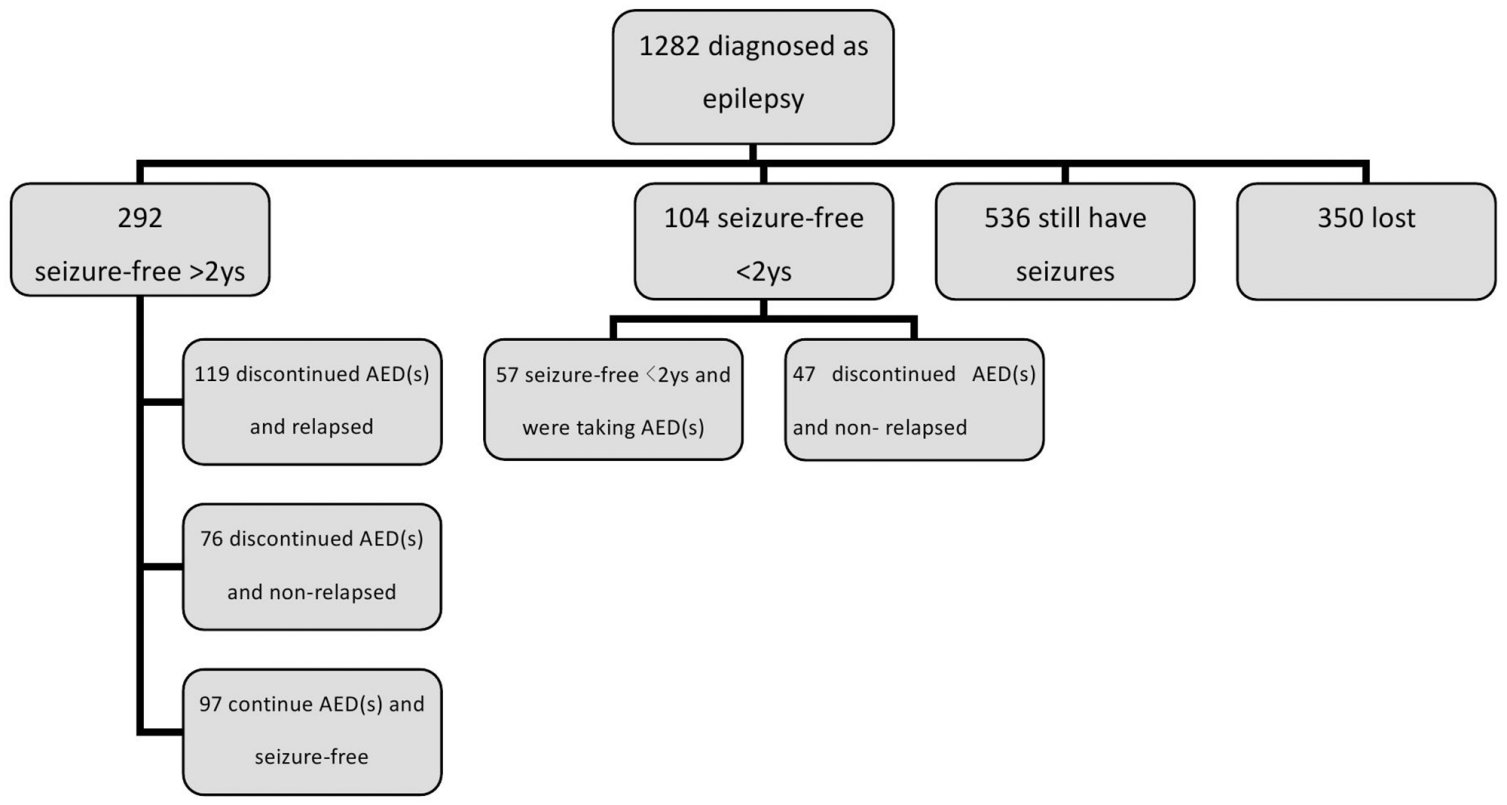
Characteristics of 1282 patients diagnosed with epilepsy.

All patients were recruited at the Department of Neurology at Huashan hospital, Shanghai, China. Between January 1, 2007 and May 31, 2011, we conducted follow-up assessments of 1282 patients from our epilepsy center database. These assessments were conducted via medical records, telephone conversations, and interviews at our outpatient clinic (Fig 1).

**Inclusion criteria were as follows:** (1) between 14 and 80 years of age, (2) history of seizures according to the 1981 classification system of the International League Against Epilepsy (ILAE1981), (3) had received continuous treatment with a stable dose of one or two different AEDs, (4) reported being seizure-free for at least 2 years prior to drug withdrawal.

**Exclusion criteria were as follows:** (1) younger than 14 or older than 80 years, (2) history of irregular AED treatment.

**Study end point (relapse criteria):** The end point of the study period coincided with seizure relapse, which was defined as the occurrence of a seizure after having at least 2 seizure-free years.

In total, 292 patients had been seizure-free for over 2 years. Of these patients, 195 had received follow-up assessments after AED withdrawal and another 97 had continued to receive AED treatment. Thus, we assessed the demographic and clinical characteristics of the patients. We used a Kaplan–Meier curve to analyze the data from the 195 patients who withdrew from AEDs.

We also used the life table method to calculate the person-years recurrence rate, as follows: Person-years recurrence rate (%) = 100*∑(persons who relapsed*year)/∑(persons in total*year)%.

We collected the following demographic and clinical information: age at onset of seizures, age at drug withdrawal, number of seizure(s) in the first 24 h, seizure frequency prior to AED treatment, course of epilepsy prior to initiation of AED treatment, age at AED treatment onset, time to achieve seizure-free status, family history of epilepsy (first- and second-degree relatives), history of febrile convulsions, history of status epilepticus, deficits revealed by neurological examinations, underlying etiology (such as craniocerebral trauma, perinatal injury, encephalitis, brain surgery), brain abnormalities revealed by neuroradiological assessment (CT/MRI), abnormal EEG features, number and type of AED treatments, length of seizure-free period prior to drug withdrawal, whether the individual received a follow-up EEG assessment following AED withdrawal, and EEG findings during follow-up assessments. We considered abnormal EEG findings to include spikes, sharp waves, polyspikes, and slow waves. We considered relevant brain abnormalities to include tumors, brain injuries, cerebral infarction, and cerebral hemorrhage.

We processed the data using the SPSS statistical package. We compared the demographic and clinical characteristics of the patients using the Student’s *t* test for continuous variables and the *χ*^*2*^ test for categorical variables between relapsed and non-relapsed groups. We used the Kaplan–Meier method to calculate the cumulative proportion of patients who relapsed after AED withdrawal. Multivariate analysis was performed by using the Cox regression model of those statistically significant variables associated with relapsed group or variables with clinical significance. We derived hazard ratios (HRs) and 95% confidence intervals (CIs) from the Cox proportional hazards model. The Cox model met the assumption of proportionality of risks. We compared the survival curves within risk factor with a log rank test.

This study was conducted in accordance with the Notes for Guidance on Good Clinical Practice from the International Conference on Harmonization as well as the Declaration of Helsinki. Huashan Institutional Review Board (HIRB) approved the relevant study protocol. All patients (or their parent or legal guardian) provided written informed consent and all minors signed a consent form.

## 3. Results

We attempted to contact 1282 patients between July 2011 and March 2012. Of these, 350 individuals did not return our calls. Of the 932 patients with whom we spoke, 292 patients (31.33%) had been seizure-free for over 2 years. Of these, 195 patients (105 females and 90 males (see Table 1) had withdrawn from AED treatment, and 119 of these individuals had relapsed (Fig 1). The mean seizure-free duration prior to AED withdrawal was 46.75±25.35 months in the non-relapsed group, and 41.58±25.10 months in the relapsed group. The general characteristics and clinical features of the patients are shown in Table 1.

**Table 1:** Demographic and clinical characteristics of patients following AED withdrawal.

Variable	All (n = 195)	a = Relapsed (n = 119)	b = Non-relapsed (n = 76)	*T*/*χ*^*2*^ value(a vs b)	*P* valu*e*
Gender(male: female)	90:105	58:61	35:41	0.13	0.71
Age($\bar{x}\pm \text{s}$, year)	29.66±13.19	29.11±12.48	30.53±14.26	0.53	0.47
Age at onset($\bar{x}\pm \text{s}$, year)	18.23±13.63	17.26±12.43	19.75±15.30	1.55	0.22
Age at drug withdrawal ($\bar{x}\pm \text{s}$, year)	25.89±13.42	24.71±12.64	27.74±14.45	2.37	0.13
Cluster seizure within first 24h(N(%))	10	8(6.1)	2(3.9)	1.60	0.21
Severity of epilepsy before onset of AED treatment					
Seizure frequency($\bar{x}\pm \text{s}$, n)	6.08±4.02	7.21±4.48	4.30±3.10	1.88	0.17
Course of disease >6 month(%)	68	49(41.5)	19(26.5)	5.34	0.021^a^
Epilepsy classification				4.81	0.090
Cryptogenic epilepsy(%)	50(28.9)	41(42.7)	29(27.3)		
Idiopathic epilepsy(%)	73(42.2)	45(49.4)	36(31.6)		
Symptomatic epilepsy(%)	50(28.9)	33(26.9)	11(17.1)		
Underlying etiology(%)	32	25(19.5)	7(12.5)	4.71	0.030 ^a^
History of febrile convulsions(%)	23	14(14.0)	9(9.0)	0.00	0.99
Family history of epilepsy(%)	8	4(4.9)	4(3.1)	0.43	0.51
Abnormal EEG findings(%)	114	70(69.6)	44(44.4)	0.016	0.90
Imaging findings(%)	31	22(19.0)	9(12.0)	1.44	0.23
Received more than one AEDs (%)	12	10(7.3)	2(4.7)	3.00	0.083^b^
Completely seizure-free after initiating AEDs(%)	106	71(64.7)	35(41.3)	-3.46	0.063 ^b^
Change in AED therapy(%)	41	20(25.0)	21(16.0)	3.27	0.070 ^b^
Without EEG follow-up	61	33(37.2)	28(23.8)	1.79	0.18
Seizure-free period before drug withdrawal ($\bar{x}\pm \text{s}$, month)	43.59±25.26	41.58±25.10	46.75±25.35	1.95	0.16

^a^ Statistically significant.

^b^ Tendency for statistical significance.

The relapse rate was 34.4 per 100 person-years. Among the 119 participants who relapsed, the probability of relapse was 23.53% during the tapering period, 21.85% at 3 months, 10.92% at 6 months, 13.45% at 1 year, 15.97% at 2 years, 7.56% at 5 years, 4.20% at 10 years, and 2.52% at 15 years.

Compared with patients in the non-relapsed group, patients with an established etiology in the relapsed group were significantly more likely to relapse (p = 0.030) (Table 1). Patients who first received antiepileptic drug treatment more than 6 months after their epilepsy diagnosis were more likely to relapse than those who received treatment within 6 months (p = 0.021).

The following patients had a higher chance of relapse: 1) patients who first received antiepileptic drug treatment more than 6 months after their epilepsy diagnosis, 2) patients with an established etiology, 3) patients who received more than one type of AED prior to drug withdrawal, 4) patients who didn't reach seizure-free immediately after initiating AED treatment, and 5) patients who made changes to their AED treatment regimen (p = 0.021, 0.030, 0.083, 0.063, and 0.070, respectively).

Other factors, such as abnormal EEG, brain abnormalities, seizure frequency prior to onset of AED treatment, history of febrile convulsions, family history of epilepsy, time to achieve seizure-free status, and absence of EEG follow-up assessments were not significantly associated with risk of relapse.

**Fig 2:**
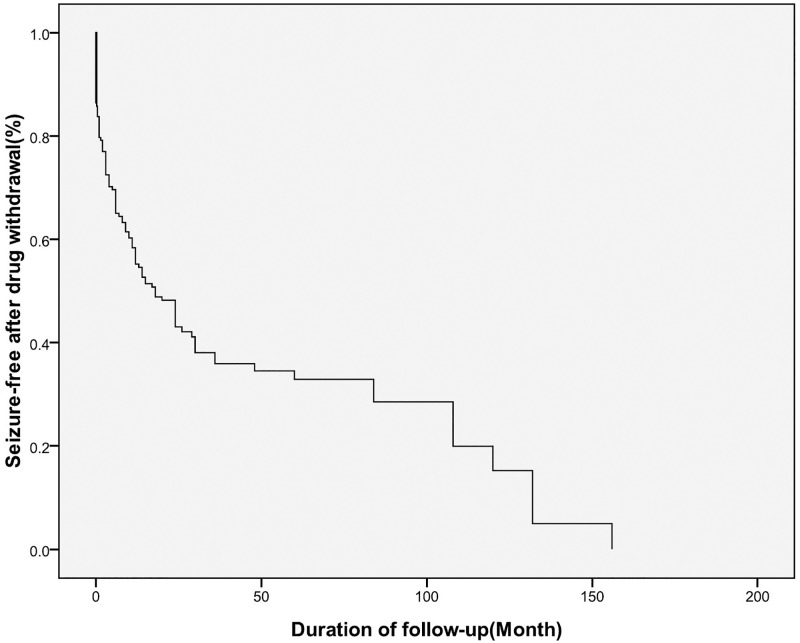
Risk of seizure recurrence after drug withdrawal: Kaplan-Meier curve.

As mentioned above, we used the Kaplan–Meier analysis to determine that 119 of the 195 patients relapsed after drug withdrawal (Fig 2). The mean period of time from AED withdrawal to relapse was 15.91±2.71 months. 76 patients did not relapse until their last visit with a clinician. The median (P_25_-P_75_) follow-up period after AED withdrawal was 24(19.8) months in the non-relapsed group. However, 22 of the patients in the non-relapsed group had a follow-up period that was shorter than 1 year.

**Fig 3:**
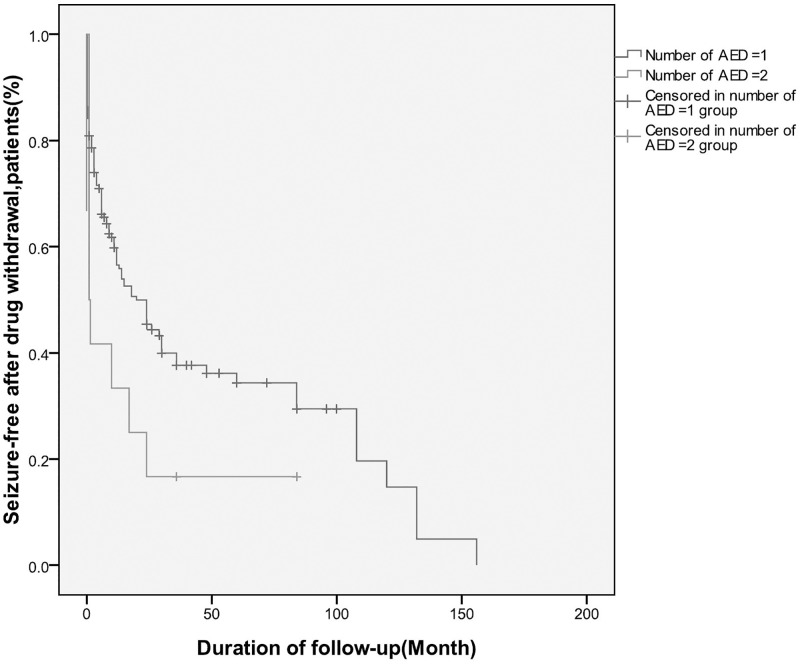
Risk of seizure recurrence after drug withdrawal: Kaplan-Meier curve(taking one more AEDs before drug withdrawal).

**Fig 4:**
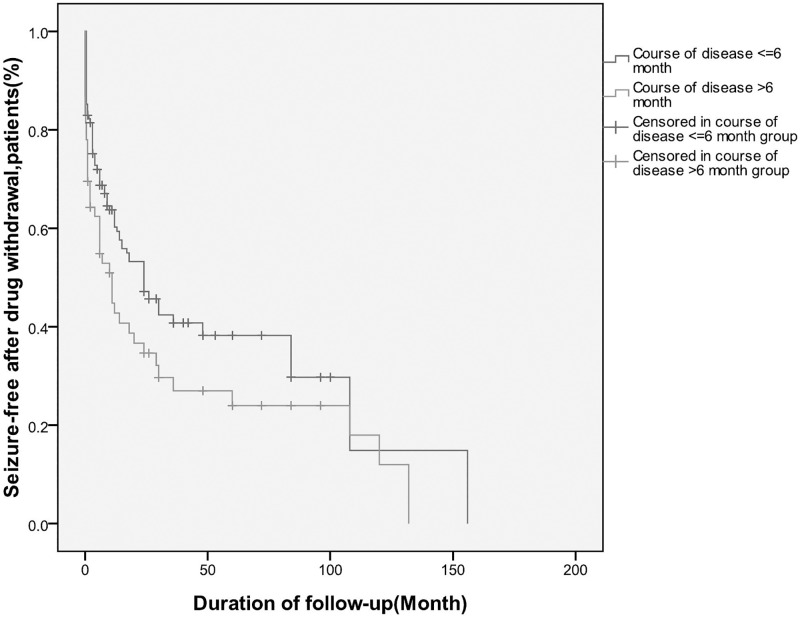
Risk of seizure recurrence after drug withdrawal: Kaplan-Meier curve(Course of disease >6 month before onset of AED).

We identified two factors that influenced seizure recurrence via Cox’s proportional hazard regression model. These were: received more than one AEDs [hazard ratio (HR) = 2.53, 95% confidence interval (95% CI) 1.24–5.16] (Table 2), and course of epilepsy (longer than 6 months) prior to initiation of AED treatment (HR = 1.47, 95% CI = 1.004–2.15) (Table 2). The log rank for number of AEDs and course of epilepsy prior to initiation of AED treatment were P = 0.008 and P = 0.046 separately (Figs 3 and 4). We also examined age at onset of seizures, age at drug withdrawal, number of seizure(s) in the first 24 h following first seizure, seizure frequency prior to AED treatment, age at AED treatment onset, time to achieve seizure-free status, family history of epilepsy (first- and second-degree relatives), history of febrile convulsions, history of status epilepticus, deficits revealed by neurological examinations, underlying etiology, brain abnormalities revealed by neuroradiological assessment (CT/MRI), abnormal EEG features, length of seizure-free period prior to drug withdrawal, and EEG findings during follow-up assessments. None of these variables were significantly associated with relapse after AED withdrawal.

**Table 2:** Risk of seizure recurrence after drug withdrawal: Cox proportional hazard ratios.

Variable	Coefficient (*β*)	Standard error	Wald *χ*^*2*^	*p*-Value	Hazard ratio	95% CI
Received more than one AEDs	0.93	0.36	6.54	0.011^a^	2.53	1.24~5.16
Course of disease >6 month before AED treatment	0.39	0.19	3.92	0.048^a^	1.47	1.004~2.15

^a^ Statistically significant.

## 4. Discussion

The aim of AED treatment is to control seizures. With optimal AED treatment, approximately 60–70% of patients reach a point where they can be considered seizure-free[3,12]. Only 31.33% (292/932) of the patients studied were seizure-free for a period of greater than 2 years. Of the initial 932 patients with whom we spoke, 292 patients had been seizure-free for over 2 years prior to AED withdrawal, while 640 patients reported a seizure-free interval of less than 2 years. Several factors may account for the low number of seizure-free individuals in our target population. First, our study population consisted of individuals who had accessed an epilepsy center. It is likely that individuals would initially access a community hospital, and be referred to an epilepsy center, if their epilepsy was difficult to control. Thus, our study population likely included patients who had faced treatment challenges. Second, it is possible that the 350 patients who did not return our calls were seizure-free and did not want to participate owing to societal stigma about illness.

We found the recurrence rate of seizures after discontinuation of AED therapy to be 34.37 per 100 person-years. This finding was consistent with that of previous studies. The reported relapse rate in patients who discontinue AED treatment ranges from 15–70%[5,8,9,13,14,15]. In our participants, recurrence of seizures mostly occurred within the first year after AED withdrawal, similar to previous reports[8,16]. We found the relapse rate to be highest within the first 3 months after AED withdrawal, after which the frequency of relapse steadily decreased.

We found that patients who received two or more types of AEDs had a relapse rate that was 2.53 (95% CI 1.24–5.16) times higher than that for patients who received a single type of AED prior to drug withdrawal. This outcome was consistent with the findings of previous studies. For instance, one study suggested that single-drug therapy decreased the chance of relapse[17]. In a recent meta-analysis, the authors found that in 8 of 23 studies, patients who received two or more types of AEDs carried a higher risk of relapse than those receiving mono-therapy[18]. Seizures are generally considered to be refractory when patients receive two or more AEDs, as these patients often have limited success with drug withdrawal, although they may achieve seizure remission for a certain period of time. One study reported that the risk of relapse after a 24-month period of seizure remission was 46.7% at 3 years for drug-resistant epilepsy patients[19]. Thus, we suggest that patients be warned that receiving two or more AEDs at the point of drug withdrawal may be associated with an increased risk of relapse.

A longer course of epilepsy and a higher seizure frequency before initiation of AED treatment have both been positively correlated with relapse risk[11]. In term of pathogenesis, seizure episodes induce axon growth, cell loss, and the formation of new synapses. A particularly prominent change is the seizure-induced sprouting of mossy fibers[20,21]. We found that seizures were more likely to recur in patients who initiated AED treatment more than 6 months after epilepsy onset. We speculate that the longer an individual waits before initiating AED treatment, the greater the severity of neuropathological changes that occur, and consequently, the greater the risk of seizure recurrence following AED withdrawal.

EEG findings are a commonly explored risk factor for relapse following treatment withdrawal. However, we did not observe a higher relapse rate in patients with abnormal EEG characteristics or in patients who did not receive an EEG follow up examination during the drug withdrawal or follow-up periods. A previous report found that patients who did not receive EEG follow-up examinations usually had poor compliance, which is related to a high recurrence rate following AED withdrawal[22]. Several studies have reported that 10–20% of seizure-free patients have abnormal EEG characteristics[2,23,24]. As mentioned above, abnormal EEG activity can predict seizure recurrence following antiepileptic drug withdrawal[25]. In post-withdrawal EEG follow-up assessments, patients with frequent seizure recurrence exhibited abnormal EEG characteristics compared with those without a high rate of seizure recurrence[16]. However, we did not find a higher risk of relapse in patients who had abnormal EEG activity before or after drug withdrawal. This discrepancy could be attributed to the large number of patients in our study who did not receive a routine EEG examination during drug withdrawal. Despite this negative result, we still suggest that EEG monitoring is important during the drug withdrawal period and emphasize the importance of EEG follow-up examinations.

We found that the following factors did not predict greater susceptibility to seizure recurrence: age at onset of epilepsy, family history of epilepsy, history of febrile convulsions, time to achieve seizure-free status, abnormal EEG findings, abnormal brain structure, established etiology, severity of epilepsy before initiation of AED treatment (seizure frequency), and age at drug withdrawal. The lack of significance of some of these probable risk factors, such as age at drug withdrawal and etiology, may be heavily influenced by recall bias.

Several limitations exist in our study. First, it was a retrospective study. This may lead to some bias of precisely collecting the information such as EEG, brain abnormalities, seizure frequency prior to onset of AED treatment, history of febrile convulsions, family history of epilepsy, course of epilepsy prior to initiation of AED treatment and classification of epilepsy syndrome. As previously reported, patients with some types of idiopathic epilepsies, such as BECTS, CAE, JAE, JME, have a good prognosis and are “time-limited”. Lack of classification of epilepsy syndrome may lead to overestimating or underestimating our results; a prospective study is needed to continue to locate prognostic risk factors. Second, the patient population was small, making it difficult for us to assess less common potential risk factors, such as symptomatic etiology and AED type.

## 5. Conclusion

Based on our findings, we suggest that patients who have been in seizure remission for more than 2 years should use caution when withdrawing from AEDs, especially when they were receiving more than one type of AED or had initiated their AED treatment more than 6 months after the initial appearance of epilepsy symptoms. Additionally, we would like to emphasize the importance of EEG follow-up examinations during drug withdrawal and during the follow-up period after drug withdrawal.

## Supporting information

S1 Fig Characteristics of 1282 patients diagnosed with epilepsy. (PDF)

S2 Fig Risk of seizure recurrence after drug withdrawal: Kaplan-Meier curve. (PDF)

S3 Fig Risk of seizure recurrence after drug withdrawal: Kaplan-Meier curve(taking one more AEDs before drug withdrawal). (PDF)

S4 Fig Risk of seizure recurrence after drug withdrawal: Kaplan-Meier curve(Course of disease >6 month before onset of AED). (PDF)

S1 Table Demographic and clinical characteristics of patients following AED withdrawal. (PDF)

S2 Table Risk of seizure recurrence after drug withdrawal: Cox proportional hazard ratios. (PDF)
