# Supplementary figures and images for: Risk factors for seizure reoccurrence after withdrawal from antiepileptic drugs in individuals who have been seizure-free for over 2 years
Source: PLoS One. 2017 Aug 1;12(8):e0181710. doi: 10.1371/journal.pone.0181710 (PMC5538662; doi:10.1371/journal.pone.0181710)

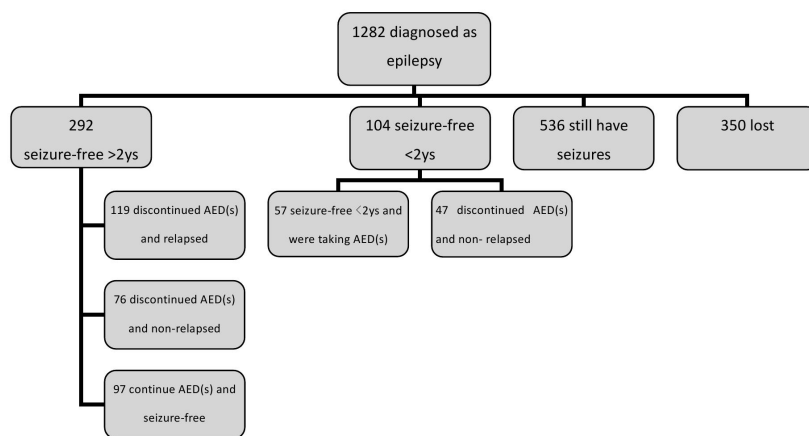

Figure 1. Characteristics of 1282 patients diagnosed with epilepsy

Supplement: S1 Fig — (PDF) [file pone.0181710.s001.pdf]

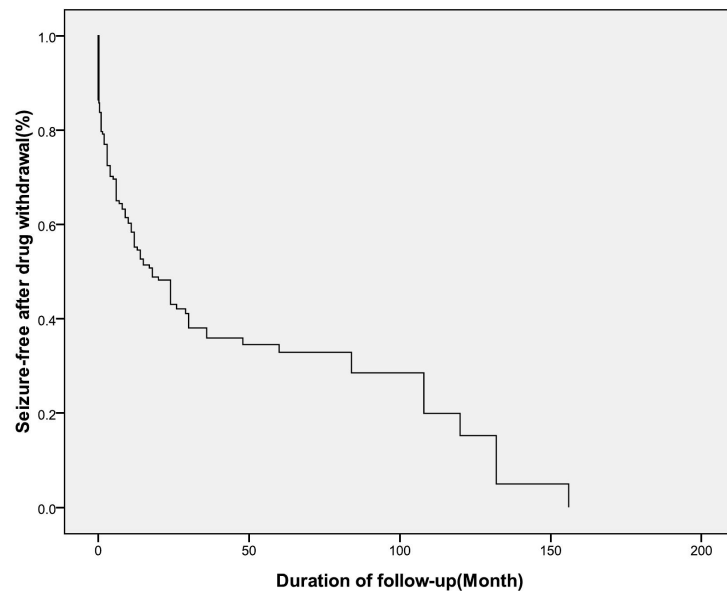

Figure 2. Risk of seizure recurrence after drug withdrawal: Kaplan-Meier curve

Supplement: S2 Fig — (PDF) [file pone.0181710.s002.pdf]
