# Supplementary material for: Risk factors for seizure reoccurrence after withdrawal from antiepileptic drugs in individuals who have been seizure-free for over 2 years
Source: PLoS One. 2017 Aug 1;12(8):e0181710. doi: 10.1371/journal.pone.0181710 (PMC5538662; doi:10.1371/journal.pone.0181710)
Supplement: S3 Fig — (PDF) [file pone.0181710.s003.pdf]

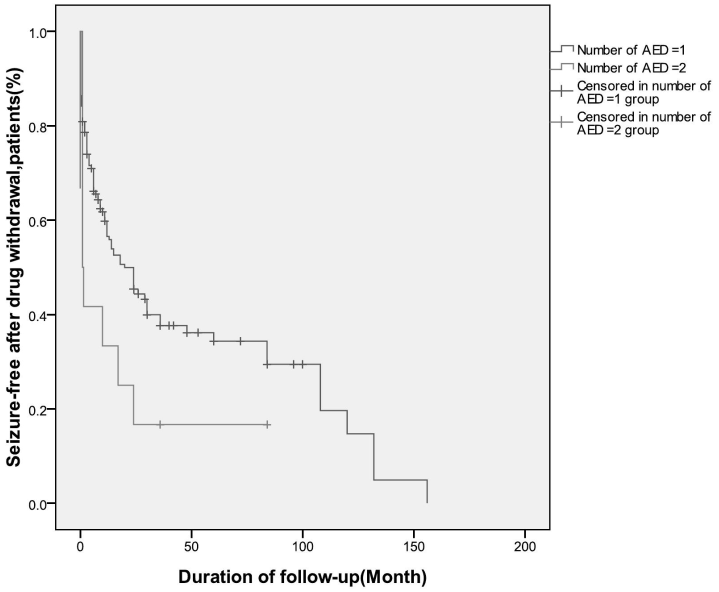

Figure 3. Risk of seizure recurrence after drug withdrawal: Kaplan-Meier curve(taking one more AEDs before drug withdrawal)
