# Supplementary material for: Risk factors for seizure reoccurrence after withdrawal from antiepileptic drugs in individuals who have been seizure-free for over 2 years
Source: PLoS One. 2017 Aug 1;12(8):e0181710. doi: 10.1371/journal.pone.0181710 (PMC5538662; doi:10.1371/journal.pone.0181710)
Supplement: S1 Table — (PDF) [file pone.0181710.s005.pdf]

Table 1 Demographic and clinical characteristics of patients following AED withdrawal

Variable	All (n=195)	a=Relapsed (n=119)	b=Non-relapsed (n=76)	T/χ^2 value(a vs b)	*P* value
Gender(male: female)	90:105	58:61	35:41	0.13	0.71
Age($\bar{x}$ ±s, year)	29.66±13.19	29.11±12.48	30.53±14.26	0.53	0.47
Age at onset($\bar{x}$ ±s, year)	18.23±13.63	17.26±12.43	19.75±15.30	1.55	0.22
Age at drug withdrawal ($\bar{x}$ ±s, year)	25.89±13.42	24.71±12.64	27.74±14.45	2.37	0.13
Cluster seizure within first 24h(N(%))	10	8(6.1)	2(3.9)	1.60	0.21
Severity of epilepsy before onset of AED treatment					
Seizure frequency($\bar{x}$ ± s, n)	6.08±4.02	7.21±4.48	4.30±3.10	1.88	0.17
Course of disease >6 month(%)	68	49(41.5)	19(26.5)	5.34	0.021[a]
Epilepsy classification				4.81	0.090
Cryptogenic epilepsy(%)	50(28.9)	41(42.7)	29(27.3)		
Idiopathic epilepsy(%)	73(42.2)	45(49.4)	36(31.6)		
Symptomatic epilepsy(%)	50(28.9)	33(26.9)	11(17.1)		
Underlying etiology(%)	32	25(19.5)	7(12.5)	4.71	0.030 [a]
History of febrile convulsions(%)	23	14(14.0)	9(9.0)	0.00	0.99
Family history of epilepsy(%)	8	4(4.9)	4(3.1)	0.43	0.51
Abnormal EEG findings(%)	114	70(69.6)	44(44.4)	0.016	0.90
Imaging findings(%)	31	22(19.0)	9(12.0)	1.44	0.23
Received more than one AEDs (%)	12	10(7.3)	2(4.7)	3.00	0.083[b]
Completely seizure-free after initiating AEDs(%)	106	71(64.7)	35(41.3)	-3.46	0.063 [b]
Change in AED therapy(%)	41	20(25.0)	21(16.0)	3.27	0.070 [b]
Without EEG follow-up	61	33(37.2)	28(23.8)	1.79	0.18
Seizure-free period before drug withdrawal ($\bar{x}$ ±s, month)	43.59±25.26	41.58±25.10	46.75±25.35	1.95	0.16

[a] Statistically significant.

[b] Tendency for statistical significance.
