# Supplementary material for: Risk factors for seizure reoccurrence after withdrawal from antiepileptic drugs in individuals who have been seizure-free for over 2 years
Source: PLoS One. 2017 Aug 1;12(8):e0181710. doi: 10.1371/journal.pone.0181710 (PMC5538662; doi:10.1371/journal.pone.0181710)
Supplement: S2 Table — (PDF) [file pone.0181710.s006.pdf]

Table 2 Risk of seizure recurrence after drug withdrawal: Cox proportional hazard ratios

Variable	Coefficient (β)	Standard error	Wald χ^2	*p*-Value	Hazard ratio	95% CI
Received more than one AEDs	0.93	0.36	6.54	0.011[a]	2.53	1.24~5.16
Course of disease >6 month before AED treatment	0.39	0.19	3.92	0.048[a]	1.47	1.004~2.15

[a] Statistically significant.
